# *Sclerotium rolfsii* Lectin Induces Stronger Inhibition of Proliferation in Human Breast Cancer Cells than Normal Human Mammary Epithelial Cells by Induction of Cell Apoptosis

**DOI:** 10.1371/journal.pone.0110107

**Published:** 2014-11-03

**Authors:** Mohammed Azharuddin Savanur, Sachin M. Eligar, Radha Pujari, Chen Chen, Pravin Mahajan, Anita Borges, Padma Shastry, Arvind. Ingle, Rajiv D. Kalraiya, Bale M. Swamy, Jonathan M. Rhodes, Lu-Gang Yu, Shashikala R. Inamdar

**Affiliations:** 1 Department of Studies in Biochemistry, Karnatak University, Dharwad, India; 2 Department of Gastroenterology, Institute of Translational Medicine, University of Liverpool, Liverpool, United Kingdom; 3 Advanced Centre for Treatment, Research and Education in Cancer, Kharghar, Navi Mumabi, India; 4 S. L. Raheja Hospital, Mahim, Mumbai, India; 5 National Centre for Cell Science, NCCS complex, Ganeshkhind, Pune, India; Children's Hospital Boston & Harvard Medical School, United States of America

## Abstract

*Sclerotium rolfsii* lectin (SRL) isolated from the phytopathogenic fungus *Sclerotium rolfsii* has exquisite binding specificity towards O-linked, Thomsen-Freidenreich (Galβ1-3GalNAcα1-Ser/Thr, TF) associated glycans. This study investigated the influence of SRL on proliferation of human breast cancer cells (MCF-7 and ZR-75), non-tumorigenic breast epithelial cells (MCF-10A) and normal mammary epithelial cells (HMECs). SRL caused marked, dose-dependent, inhibition of proliferation of MCF-7 and ZR-75 cells but only weak inhibition of proliferation of non-tumorigenic MCF-10A and HMEC cells. The inhibitory effect of SRL on cancer cell proliferation was shown to be a consequence of SRL cell surface binding and subsequent induction of cellular apoptosis, an effect that was largely prevented by the presence of inhibitors against caspases -3, -8, or -9. Lectin histochemistry using biotin-labelled SRL showed little binding of SRL to normal human breast tissue but intense binding to cancerous tissues. In conclusion, SRL inhibits the growth of human breast cancer cells via induction of cell apoptosis but has substantially less effect on normal epithelial cells. As a lectin that binds specifically to a cancer-associated glycan, has potential to be developed as an anti-cancer agent.

## Introduction

Lectins are a group of highly diverse proteins of non-immune origin ubiquitously distributed in plants, animals and fungi. A lectin contains at least one non-catalytic domain that selectively recognizes and reversibly binds to specific glycans [1]. Some lectins can recognize tumour associated-glycans and are therefore useful tools to differentiate malignant from benign tumours and also to study cancer-associated glycosylation changes [2]. Aberrant glycosylation in cancerous and pre-cancerous tissues is common and this is exemplified by incomplete synthesis of carbohydrate chains, allowing higher expression of precursor carbohydrate moieties, such as the oncofetal Thomsen-Freidenreich [CD176: Galβ1, 3GalNAcα-O-Ser/Thr, TF] and Tn [CD175: GalNAcα-O-Ser/Thr] antigens whose expressions are correlated with tumor progression and metastasis [3], [4], [5]. Recent studies have shown the exclusive expression of *N*-glycolylneuraminic acid (Neu5Gc) on glycoproteins and gangliosides in human melanoma, colon, retinoblastoma and breast cancers but not in normal tissues [6], another example is that of a monosialicganglioside, *N*-glycolyl GM3, which is not expressed in normal human tissues, but is detected in human breast tumors [7]. Hence these tumour-associated glycans are gaining significance as attractive candidates for the development of anti-cancer strategies [8].

*Sclerotium rolfsii*, a soil-borne phyto-pathogenic fungus, secretes a developmentally regulated lectin [9], SRL, which has been shown previously to recognizes the oncofetal TF antigen and its derivatives [10]. The crystal structure of SRL, both in its free form and as complex with *N*-acetyl-D-galactosamine (GalNAc) and *N*-acetyl-D-glucosamine (GlcNAc) has been determined at 1.1 Å, 2.0 Å and 1.7 Å resolutions, showing the presence of two carbohydrate binding-sites per SRL monomer. The primary carbohydrate-binding site of SRL recognizes the TF disaccharide and *N*-acetyl-D-galactosamine and the secondary binding site recognizes *N*-acetyl-D-glucosamine [11]. Our previous studies have shown that SRL binds to the surface of cancer cells and inhibits the growth of human colon and ovarian cancer cells *in vitro* and suppresses growth of colon xenografts *in vivo* [12], [13]. The present study investigated the effect of SRL on proliferation of human breast cancer (MCF-7 and ZR-75), which are known to express Thomsen–Friedenreich (T/TF) antigen and its derivatives due to reduced expression of core-2 β1,6-GlcNAc-transferase [14] and normal mammary (HMECs and MCF-10A) epithelial cells in order to explore its possible application as a selective anticancer drug.

## Materials and Methods

### Materials

BSA (Bovine serum albumin), bovine sub maxillary mucin and Calcein AM (Acetoxy Methyl) fluorescent dye, were obtained from Sigma Chemical Co. (St. Louis, USA). FCS (Fetal calf serum) was from Gibco Invitrogen (Paisley, UK), 3-3' diaminobenzidine chromogen/H_2_O_2_ substrate in buffered solution (pH 7.5) (DAB kit) was obtained from Bangalore Genei, Bangalore, India. Hybond poly vinylene diflurodine (PVDF) membrane and MTT [3-(4, 5-dimethylthiazol-2-yl)-2,5-diphenyltetrazolium bromide] were obtained from GE Life Sciences (Pittsburgh, PA, USA). Caspase Glo3/7 Assay kit was procured from Promega, Madison, USA and caspase inhibitors, Caspase-3 z-VAD (OMe) (N-Benzyloxycarbonyl-Val-Ala-Asp), caspase-8 z-IETD (Ile-Glu(O-Me)-Thr-Asp(O-Me)), caspase-9 Z-LEHD (Leu-Glu-(OMe)-Thr-Asp-(OMe)), were from Calbiochem, Nottingham, UK. Annexin-V detection kit was procured from Biovision (USA). Antibodies against active caspase-3 were from Epitomics (USA). Polyclonal mouse antibodies to FasL (Fas Ligand), FADD (Fas-associated death domain), Caspase-8, -9, t-BID (Truncated BH3 interacting-domain death agonist) were procured from Santa Cruz Biotechnology, California, USA. Mouse polyclonal PARP (Poly ADP ribose polymerase) antibody was from PIERCE, Barrington, USA. Species-specific HRP (Horseradish peroxidise)-labelled secondary antibodies were procured from Bio Rad, Hercules, USA. aBSM (Asialo bovine sub maxillary mucin) and asialo glycophorin A was prepared by acid hydrolysis of bovine sub maxillary mucin and glycophorin A, according to the method of R.G. Spiro [15].

### Cell culture

The human breast cancer cells MCF-7 and ZR-75 were obtained from the European Cell Culture Collection via the Public Health Laboratory Service (Porton Down, Wiltshire, UK) and cultured in DMEM (Dulbecco's modified Eagle's medium) supplemented with 10% FCS, 100 units/ml penicillin, 100 µg/ml streptomycin (complete DMEM) at 37°C in 5% CO_2_. Human Mammary Epithelial Cells (HMEC) derived from reduction mammoplasty were purchased from Lonza (Walkersville, MD, USA) and were cultured in Mammary epithelial basal media (MEBM) containing necessary supplements of Bovine pituitary extract (BPE), Human epidermal growth factor (hEGF), Human insulin, Hydrocortisone, Gentamicin (30 mg/ml) and Amphotericin (15 mg/ml). Non-tumorigenic MCF-10A cells derived from human fibrocystic mammary tissue were a kind gift from Dr. Milind Viadya and were cultured in DMEM-F12 (1∶1) complete media containing necessary supplements of EGF (20 ng/ml), Hydrocortisone (0.5 mg/ml), Cholera toxin (100 ng/ml), Insulin (10 µg/ml), penicillin (100 units/ml) and streptomycin (100 µg/ml) and maintained at 37°C in 5% CO_2_.

### SRL conjugation with FITC, biotin and Sepharose-4B

SRL from the sclerotial bodies of the fungus was purified as described previously [6]. SRL was conjugated with FITC (Fluorescein isothiocyanate) by the method described by Goldman et al. [16]. Biotinylation of SRL was carried out according to the method of Duk et al. [17]. SRL was conjugated to Sepharose-4B by the method of March et al. [18]. SRL conjugated Sepharose-4B was suspended in TBS (25 mM Tris buffered saline, pH 7.5) and stored at 4°C.

### Lectin Histochemistry

Human breast tissue samples (normal, primary and metastatic cancer tissues) used in the study were procured from S. L. Raheja Hospital, Mumbai, India, consent was not obtained from donors for the use of tissue and Institutional Review Board of S. L. Raheja Hospital, Mumbai, India (IRB No. 08/2009) has approved the method of consent, prior to conducting this study. Tissue samples were obtained during surgery, fixed in buffered formalin and embedded in paraffin for routine pathological examination by Haematoxylin-Eosin staining. All the samples used for histochemistry were retrospectively retrieved from the archives where samples were stored with appropriate histopathology numbers. Since there was no clinical intervention in the approved study, the ethic committee granted permission to retrieve paraffin blocks and slides for analysis. None of the authors were involved in sample collection and they did not have access for sample collection.

Lectin histochemistry using biotinylated-SRL (20 µg/ml) was carried out on 5 µm tissue sections as described by Boland et al [19]. SRL binding was evaluated by optical analysis and scored by measuring the mean area of stained cells and expressed as intense (+++), moderate (++), light (+) and no binding (−).

### Assessment of cell proliferation

Sub-confluent MCF-7 and ZR-75 cells were seeded at 4×10^4^ cells/ml in 96 well plates in DMEM complete medium for 48 h. The medium was replaced with serum-free DMEM containing 0.5% BSA (w/v) and the cells were incubated with various concentrations of SRL (0–40 µg/ml), BSA (40 µg/ml) and TBS for different time intervals (24, 48 and 72 h) in the presence or absence of TF-expressing glycoproteins (asialo bovine submaxillary mucin and asialoglycophorin-A). MCF-7 cells were treated with SRL (20 and 40 µg/ml with a hemagglutination titre of 128 and 256 respectively) and Sepharose-conjugated SRL with equivalent hemagglutination titre of 128 and 256 at 37°C for 72 h. In another experiment MCF-7 cells were treated with SRL for 48 h with or without pre-incubation with Pan caspase-inhibitor [Z-VAD (OMe), 50 µM]. The cells processed as above were then labelled with 10 µg/ml Calcein AM at 37°C for 30 min in 5% CO_2_, lysed with 100 µl lysis buffer (50 mM Tris-HCl, pH 6.8, containing 5% SDS (Sodium dodecyl sulphate) and 2% mercaptoethanol) and the fluorescent intensity was read using a Tecan i-200 microplate reader with excitation wavelength of 485 nm and emission wavelength of 535 nm.

Sub-confluent HMECs and MCF-10A cells were seeded at 4×10^4^ cells/ml in 96 well plates in MEBM or DMEM-F12 media respectively with appropriate supplements for 48 h. In the case of MCF-10A the media were then replaced with serum-free supplemented DMEM-F12 containing 0.5% BSA (w/v) and both cell lines were then incubated with various concentrations of SRL for different time intervals (24, 48 and 72 h). MCF10A cells were labeled with Calcein AM and the fluorescent intensity was measured as described above. HMECs viability was measured by MTT (3-(4,5-dimethylthiazolyl)-2,5-diphenyltetrazolium bromide) assay. Briefly, HMECs were cultured in MEBM for 48 h and incubated with various concentrations of SRL for 48 h. Following incubation, 10 µl of MTT (5 mg/ml) was added to each well followed by lysis of the cells in 100 µl of 10% SDS in 0.01 N HCl and absorbance was measured at 570 nm, with reference wavelength of 640 nm, using ELISA plate reader. Percentage viable cell number was calculated, with respect to controls considered as 100%.

### Assessment of SRL cell surface binding

MCF-7 and HMEC cells (0.5×10^6^ cells/ml) were grown on cover slips in DMEM and MEBM media respectively and incubated with 3% BSA for 1 h at 4°C. Cells were washed with PBS (Phosphate buffered saline) and incubated with FITC-SRL (2 µg/ml) for 1 h at 4°C. The cells were washed thrice with PBS and fixed with 2% para-formaldehyde for 10 min. The cells were counter-stained with DAPI (4',6-diamidino-2-phenylindole) before visualization using a Confocal Laser scanning Microscope (Zeiss LSM 510, Germany) equipped with 488 nm and 560 nm Argon lasers.

### Assessment of cellular apoptosis

#### Detection of Apoptosis by Annexin-V/PI staining

MCF-7 cells (0.5×10^6^) cultured in 6 well plates were treated with or without SRL (20 µg/ml) for 24, 36, and 48 h in serum free media and then harvested by gentle trypsinization, washed with PBS and resuspended in binding buffer from Biovision Annexin–V kit. The resuspended cells were incubated with 5 µl FITC Annexin-V (Biovision) and 5 µl PI (Propidium Iodide) for 5 min at 37°C in the dark before analysis by flow cytometry (FACS Calibur, BD). The percentages of cells positive for Annexin V, PI alone and both Annexin V and PI were calculated by dot blot analysis using Cell Quest Pro software (BD Biosciences).

#### Caspase 3/7 activity

MCF-7 cells were cultured in 96 well plates at 4×10^4^ cells/ml in DMEM complete media for 48 h before introduction of caspases inhibitors (50 µM) against caspase-3(z-VAD-(OMe)), caspase-8 (z-IETD) or caspase-9 (z-LEHD) in serum-free media followed by addition of SRL (20 µg/ml) for 48 h. Cell apoptosis was then determined by measuring caspase-3/7 activity using Caspase Glo3/7 assay according to manufacturer's instructions.

In another experiment MCF-10A cells were cultured in 96 well plates at 4×10^4^ cells/ml in DMEM-F12 complete media for 48 h before treatment with SRL (20 µg/ml) for 48 h. Cell apoptosis was then determined by measuring caspase-3/7 activity using Caspase Glo-3/7 assay according to manufacturer's instructions.

In another experiment MCF-7 cells (0.5×10^6^) were grown on cover slips in 6 well plates in DMEM complete media and incubated with SRL 20 µg/ml for 36 h followed by staining with FITC-SRL (2 µg/ml), Anti-active caspase-3 primary antibody followed by pycoerythrin labelled secondary antibody and images were observed by fluorescence microscopy.

#### Cell cycle analysis

MCF-7 cells (0.5×10^6^) were cultured in 6 well plate and treated with or without SRL (20 µg/ml) for 24, 36, and 48 h in serum-free media before harvesting by gentle trypsinization, followed by washing with PBS, fixation in 70% chilled ethanol and were then kept at −20°C until further analysis. Cells were washed and rehydrated in PBS, treated with 50 µl Ribonuclease A (5 mg/ml in PBS, DNase free) for 10 min at room temperature and stained with Propidium Iodide (50 µg/ml in PBS) for 30 min at 37°C. Data acquisition was performed using the FL-2A channel of the Flow Cytometer (FACS Calibur, BD) and was analyzed by Mod Fit LT mac2.0 software (Verity) for the distribution of cells in different phases.

#### Western Blotting

MCF-7 cells were incubated with or without SRL (20 µg/ml) in serum-free media at 37°C for 24, 36 or 48 h. The cells were harvested by gentle trypsinization and lysed using RIPA lysis buffer (120 mM NaCl, 1.0% Triton X-100, 20 mM Tris–HCl, pH 7.5, 100% glycerol, 2 mM EDTA, and protease inhibitor cocktail, Roche, Germany). Total protein was fractionated on SDS-PAGE and blotted onto Hybond polyvinylidene difluoride membranes. After blocking with 5% BSA the blots were incubated with antibodies against FasL, FADD, Caspase-8, tBID, caspase -9, active caspase-3, or PARP for 4 h followed by incubation for 1 h with the species specific HRP conjugated secondary antibodies (1∶1000). The blots were developed and visualized by chemiluminescence using ECL plus substrate (Amersham, GE). The blots were stripped and reprobed for β-actin for protein loading control.

### Statistical analysis

Each experiment was performed at least three times, and each time in triplicate. Results were analyzed by one-way ANOVA followed by ‘Newman-Keuls’ multiple comparisons using Stat Direct software and data considered significant (*) when P≤0.05.

## Results

### SRL shows strong binding to cancer tissues

**Figure 1:**
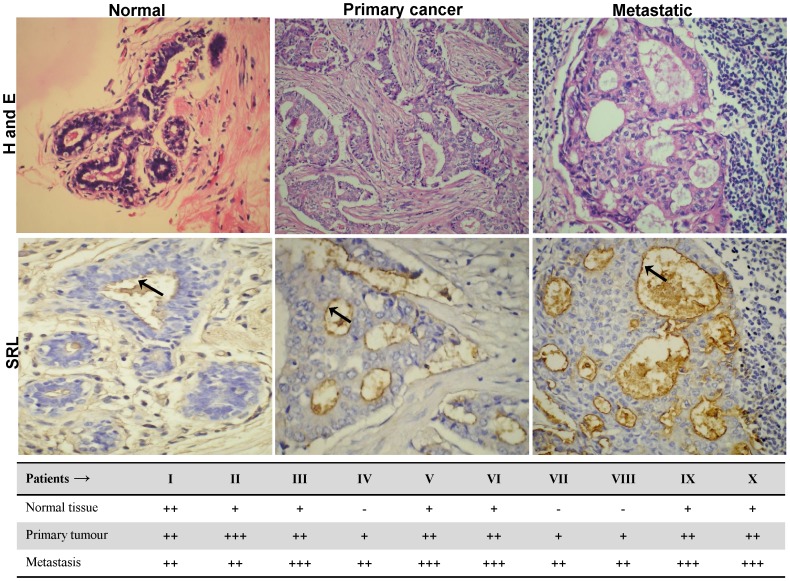
SRL histochemistry to normal and cancerous human breast tissues. SRL histochemistry was performed in human breast (normal, primary cancer and metastatic) tissue samples. SRL shows weak binding to normal human breast tissues but strong binding to primary cancer and metastatic breast tissues. All the images were obtained with 100X magnification. Arrows point to SRL binding to apical surface of the secretory gland epithelia. Representative images of both Haematoxylin-Eosin and Biotin-SRL staining are shown. SRL binding was evaluated through optical analysis by measuring the mean area of stained cells scored arbitrarily as intense (+++), moderate (++), light (+) and no staining (−).

SRL showed intense staining in primary and metastatic human breast cancer tissues and relatively light staining in the normal tissues (Figure 1). This is in keeping with the binding specificity of SRL to the TF-related glycans which are known to be over-expressed in cancerous and in particular metastatic tissues.

### SRL shows strong inhibition of proliferation in human breast cancer cells

**Figure 2:**
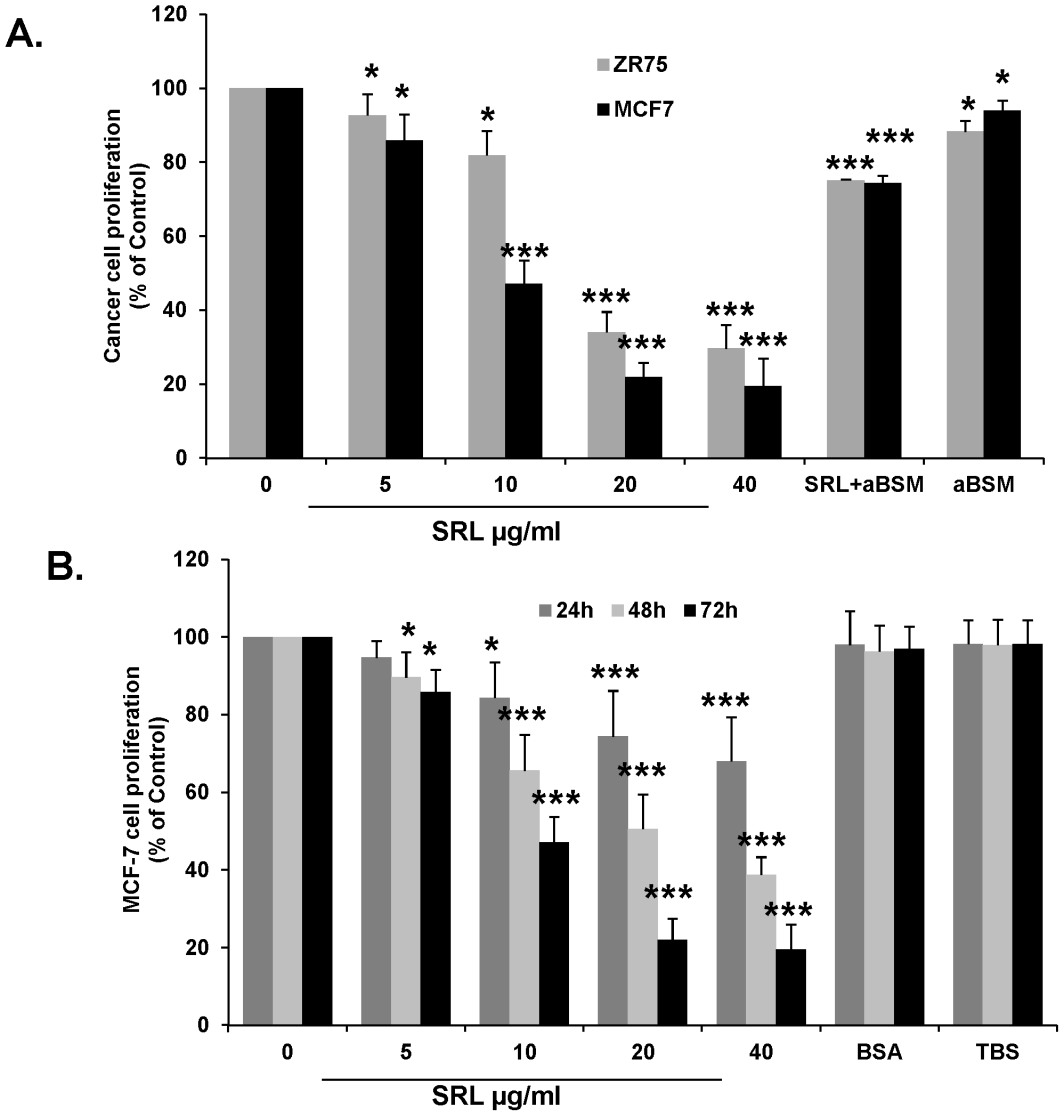
SRL inhibits proliferation of human breast cancer cells. (**A**). SRL causes dose-dependent inhibition of human breast cancer MCF-7 and ZR-75 cell proliferation. The cells were incubated with or without different concentrations of SRL for 72 h. SRL-mediated cell growth inhibition is prevented by the presence of TF-expressing glycoprotein: MCF-7and ZR-75 cells were incubated with or without 20 µg/ml SRL in the presence of 100 µg/ml asialo bovine submaxillarymucin (aBSM) for 72 h. SRL caused time-dependent inhibition of MCF-7 cell proliferation. (**B**). The MCF-7 cells were incubated with or without different concentrations of SRL, BSA (40 µg/ml) and TBS for 24, 48 and 72 h before cell proliferation was assessed. Data represent Mean ±SD of triplicate determinations from three different assessments. *p<0.05; ***p<0.001.

The presence of SRL was found to cause a dose- and time-dependent inhibition of proliferation of human breast cancer MCF-7 and ZR-75 cells. At 20 and 40 µg/ml SRL caused 49.5±6.5% (*P<0.0001*) and 61.3±2.7% (*P<0.0001*) growth inhibition in MCF-7 cells after 48 h and 78.1±3.8% (*P<0.0001*) and 80.6±7.6% (*P<0.0001*) inhibition after 72 h, whereas the presence of SRL at 20 and 40 µg showed 66±3.8% (P<*0.0001*) and 70.4±7.4% (P<*0.0001*) inhibition of ZR-75 cells after 72 h (Figure 2A and B). The inhibitory effect of SRL (20 µg/ml) on proliferation of MCF-7 and ZR-75 cells was blocked by the presence of TF-expressing aBSM (100 µg/ml) by 66.28±0.25%(P<*0.0001*) and 62.33±2.0% (*P<0.0001*) respectively and also by asialo-glycophorin A in MCF-7 cells, suggesting that the growth inhibitory effect of SRL is mediated by its carbohydrate binding sites.

**Figure 3:**
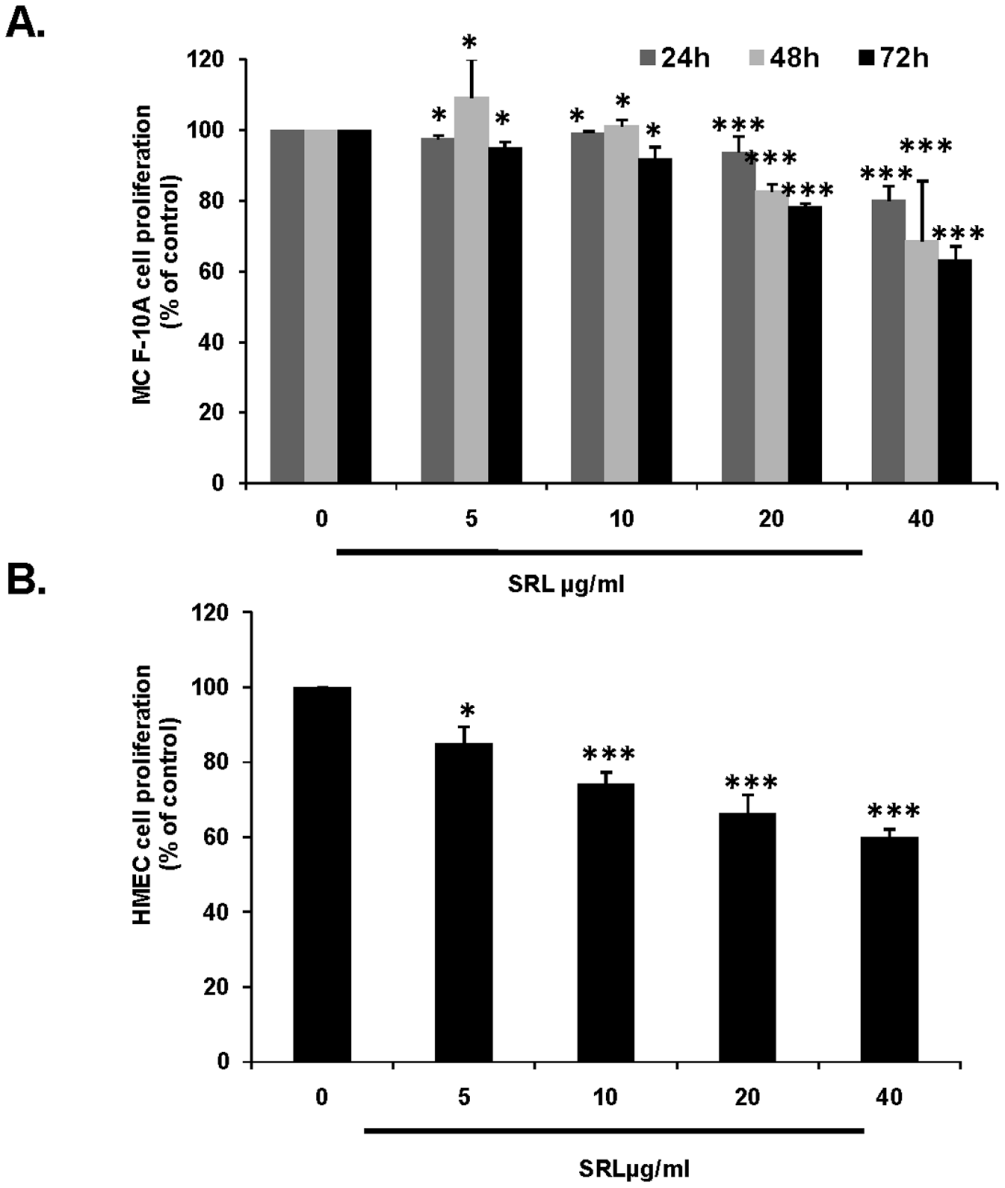
Effect of SRL on proliferation of non-tumorigenic and normal human breast epithelial cells. (**A and B**) Non-tumorigenic MCF-10A and normal HMECs were incubated with different concentrations of SRL in serum free media for different time intervals (24, 48 and 72 h for MCF-10A) and 48 h (HMECs). Cell proliferation was measured using Calcein AM and MTT for MCF-10A and HMEC respectively. The experiments were carried out in triplicate, and the data were expressed as Mean ± SD percentage of control. *p<0.05; ***p<0.001.

It was found that the presence of SRL caused weak inhibition of proliferation of non-tumorigenic MCF-10A cells derived from human fibrocystic mammary tissue and also of normal breast epithelial HMEC cells. After 24, 48 and 72 h incubation with the cells, SRL at 40 µg/ml showed inhibition of MCF-10A cell proliferation by 20±3.1% (P<0.0001), 31.5±1.0% (P<0.0001) and 36.7±4.2% (P<0.0001) respectively. A similarly weak growth-inhibitory effect of SRL (at 40 µg/ml) on HMECs was observed after 48 h by 40.0±2.2% (P<0.0001) (Figure 3A and B). The results indicate that in comparison to its strong growth inhibitory effect in cancer cells, SRL has only weak effect on normal epithelial cells. A four-time greater concentration of SRL is required to produce similar growth inhibition in normal epithelial cells in comparison with breast cancer cells (Figure 3).

### SRL-mediated growth inhibition is a direct consequence of its cell surface binding

**Figure 4:**
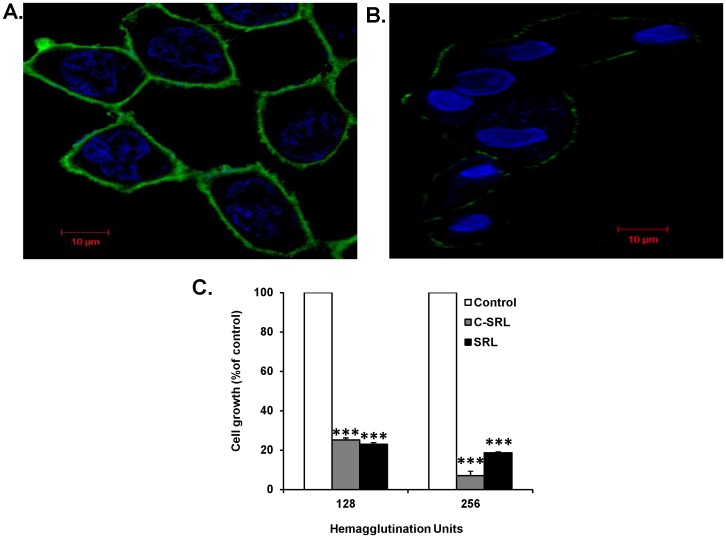
Sepharose-conjugated SRL induced growth inhibition of breast cancer cells. (**A**) SRL shows strong binding to MCF-7 cells and very weak binding to (**B**) HMEC. (**C**) MCF-7 cells were incubated without or with SRL or Sepharose-SRL (C-SRL), at equivalent concentrations to SRL with 128 and 256 hemagglutination units (HAU) activity for 72 h before the cell proliferation was assessed by Calcein AM. Data represent Mean ±SD of triplicateexperiments. *** p<0.0001 when compared to control.

SRL showed a strong and uniform binding to MCF-7 cells (Figure 4A) where as weak and uneven binding to HMECs (Figure 4B). Treatment with Sepharose-conjugated SRL demonstrated a similar growth inhibitory effect on MCF-7 cells as the unconjugated form (Figure 4C). This indicates that binding of SRL to the cell surface is sufficient to trigger its growth inhibitory effect.

### SRL-mediated cell growth inhibition is associated with induction of cell apoptosis

**Figure 5:**
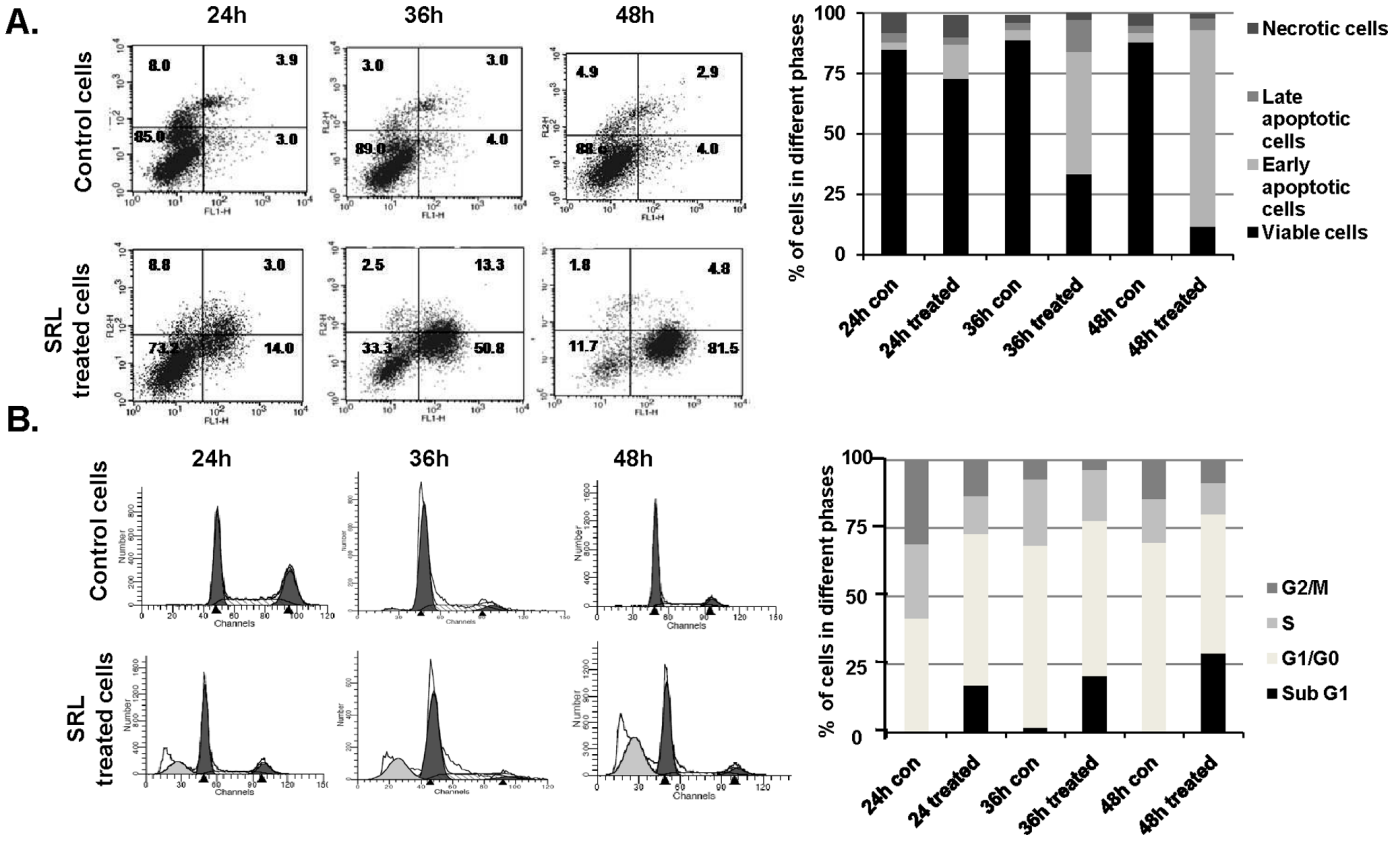
SRL induces cell apoptosis. (**A**) Annexin V cell surface binding in MCF-7 cells after incubation with SRL (20 µg/ml) for 24, 36 or 48 h. Percentages of cells in each quadrant are shown as inserts. The graph indicates percentage of the cell population in different phases. (**B**) Effect of SRL on cell cycle. MCF-7 cells were exposed to SRL (20 µg/ml) for 24, 36 and 48 h before the cellswere stained with propidium iodide and DNA content was measured by flow cytometry. The graph indicates percentage of cells in subG1, G1, S, and G2–M phases of the cell cycle.

**Figure 6:**
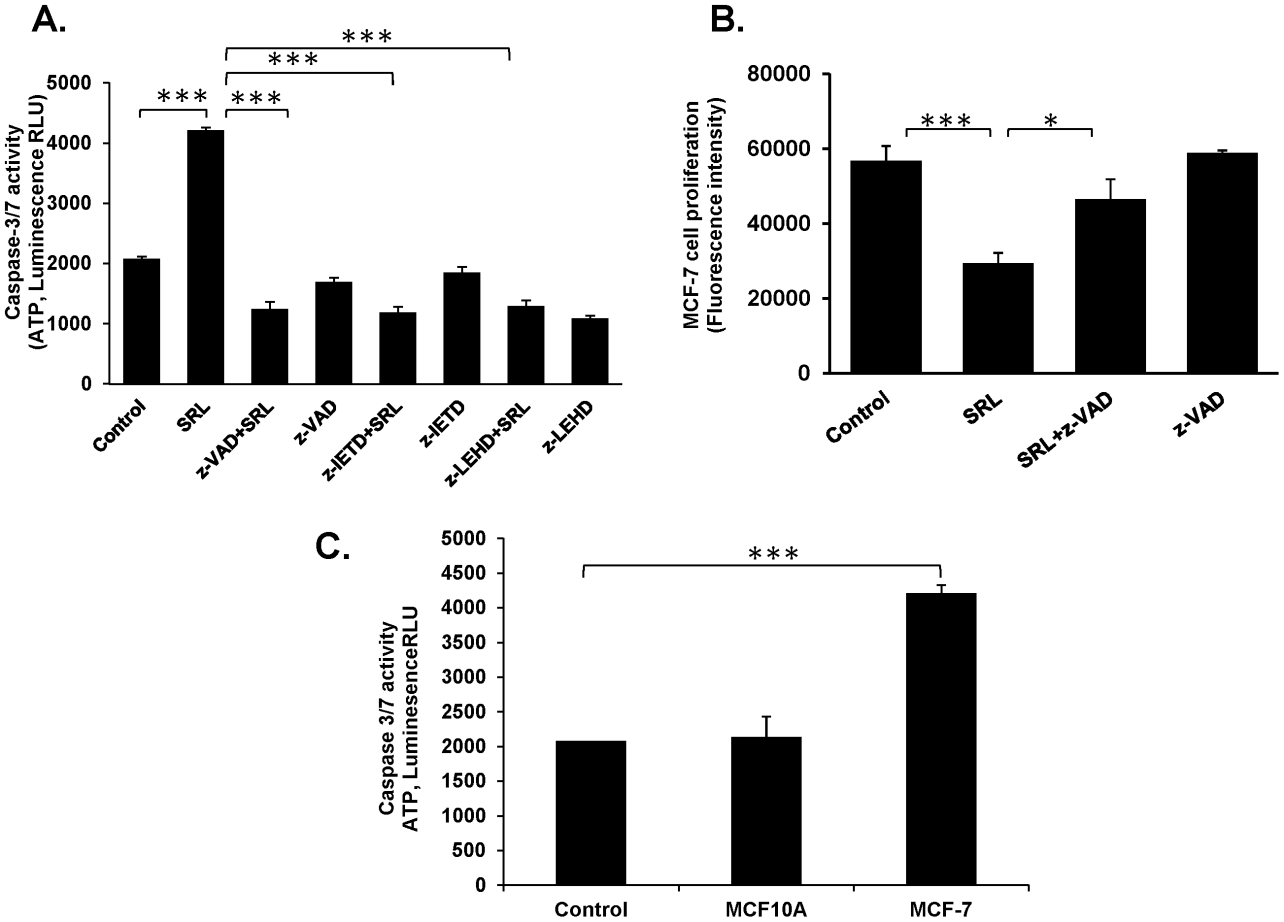
SRL-mediated inhibition of cell proliferation is associated with induction of apoptosis. (**A**) MCF-7 cells were incubated without or with SRL (20 µg/ml) in the presence or absence of inhibitors (50 µM) to caspase-8 (z-IETD), caspase- 9 (z-LEHD) or pan-caspases (z-VAD-(OMe) for 48 h before the caspase 3/7 activity was assessed. (**B**) MCF-7 cells were incubated with or without SRL (20 µg/mL) in the presence or absence 50 µM pan-caspase inhibitor z-VAD-(OMe) for 48 h before the viable cells were assessed using Calcein AM. *P<0.05, **P<0.01, ***P<0.001. (**C**) MCF-10A and MCF-7 cells were incubated without or with SRL (20 µg/ml) for 48 h before the caspase 3/7 activity was assessed by caspase 3/7 Glo assay. P = 0.5847 and ***P<0.001 respectively for MCF-10A and MCF-7 cells.

**Figure 7:**
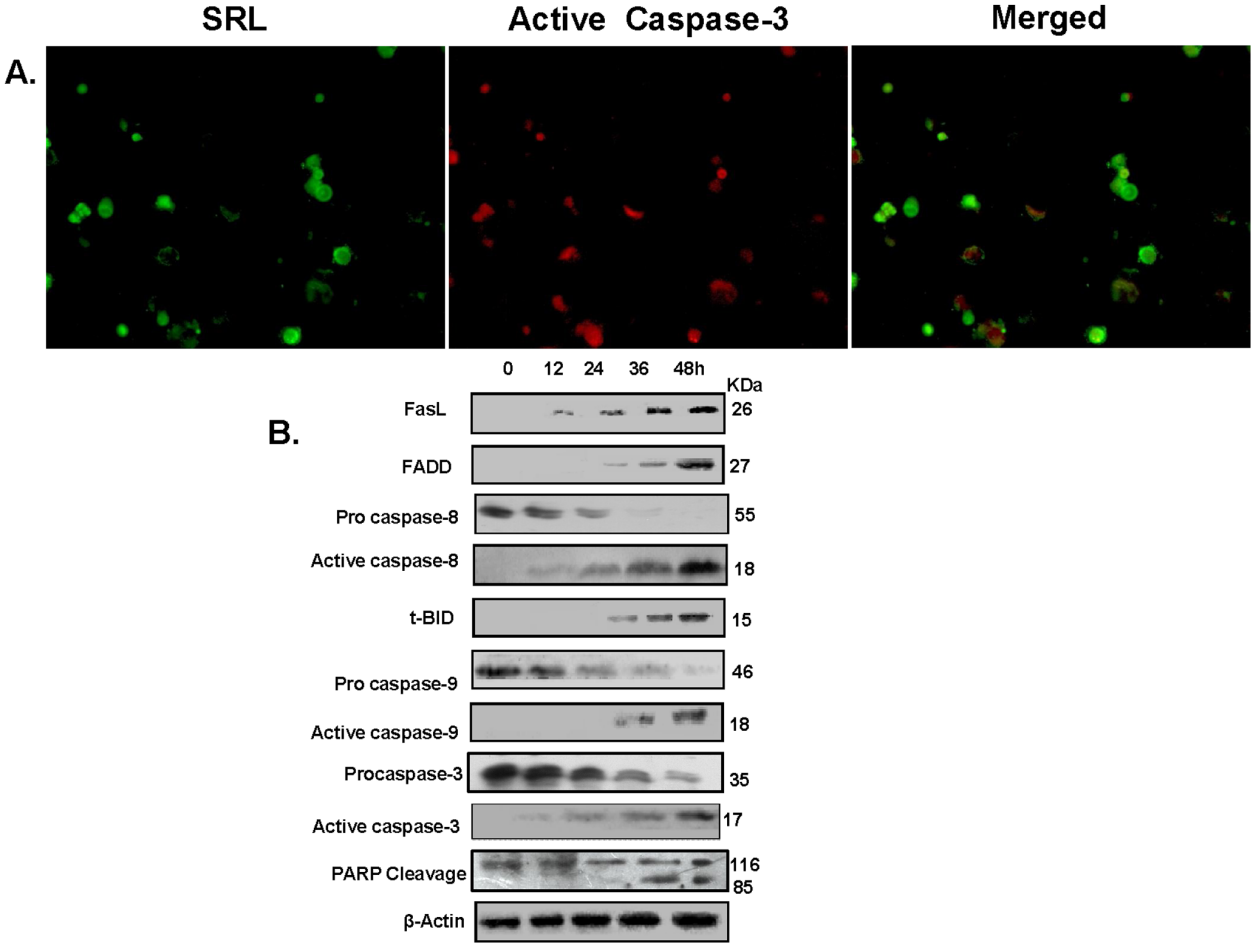
SRL induced apoptosis involves alteration of regulators. (**A**) Immunohistochemistry of MCF-7 cells after treatment with SRL. MCF-7 cells were grown on cover slips and incubated with 20 µg/ml SRL for 36 h followed by staining with FITC-SRL, Anti-active caspase-3 primary antibody followed by pycoerythrin labelled secondary antibody and images were observed by fluorescence microscopy. (**B**) SRL-induced apoptosis in MCF-7 cells involves alteration of regulators in both the extrinsic and intrinsic apoptosis pathways. MCF-7 cells were incubated with SRL (20 µg/ml) for different time intervals. At specific time intervals whole cell protein was obtained, separated by electrophoresis, blotted on to Immobilon polyvinylidene difluoride membrane and probed with antibodies against FasL, FADD, tBID, PARP, pro-caspase-8, -9,-3 or active caspase-8, -9,-3. The bands were visualized using chemiluminescence kit. The blots were stripped and reprobed for β-actin which was used as loading control.

To assess whether the growth inhibitory effect of SRL is related to induction of cell apoptosis, Annexin-V cell surface binding, existence of sub-G1 population and cellular caspase-3/7 activity were measured after cell treatment with SRL. MCF-7 cells treated with SRL for 24, 36 and 48 h showed a gradual increase in population of early apoptotic cells (Annexin V positive and PI negative), by 5-fold, 13-fold and 21-fold, respectively, as compared with untreated cells (Figure 5A). SRL treatment also increased the cell sub-G1 population by 18, 23 and 39% after 24, 36 and 48 h respectively in comparison with the control cells (Figure 5B). Furthermore, treatment of MCF-7 cells with SRL (20 µg/ml) for 48 h resulted in 2.02-fold (P<0.001) increase in cellular caspase-3/7 activity in comparison to the untreated cells (Figure 6A). On the other hand, treatment with the same concentration of SRL (20 µg/ml) for 48 h showed no significant effect on caspases-3/7 activity of non-tumorigenic MCF-10A cells (Fig. 6C). The presence of inhibitors against caspase-8 or caspase-9 in the culture showed to prevent SRL-induced caspase-3/7 activation. The presence of pan-caspase inhibitor also showed to greatly reduce the growth-inhibitory effect of SRL on MCF-7 cells (Figure 6B). Activation of caspase-3 was further evidenced by images of fluorescence microscopy, where SRL treated MCF-7 cells showed positive staining for caspase-3 antibody (Figure 7A). All these results collectively suggest that SRL-mediated cell growth inhibition is linked to its induction of cell apoptosis; an effect that probably involves the activation of both the intrinsic and extrinsic apoptotic pathways. This conclusion is supported by the increased expressions of activated caspases-8, -9 and -3 and decreased expressions of the inactive form of each caspase and also PARP cleavage in a time-dependent manner after introduction of SRL. The time-dependent increase in the expressions of FasL, FADD, and truncation of Bid (Figure 7B) are also in keeping with the likely involvement of both the intrinsic and extrinsic apoptotic pathways in SRL-mediated apoptosis.

## Discussion

This study shows that the presence of SRL caused strong inhibition of proliferation in human breast cancer MCF-7 and ZR-75 cells but a much lesser effect was observed in non-tumorigenic human mammaryMCF-10A and normal HMEC cells. The effect of SRL on cell growth inhibition is found to be linked to SRL cell surface binding and subsequent induction of cell apoptosis. SRL also shows strong binding to cancerous and metastatic tissues but very weak binding to normal human breast tissues.

Apoptosis or programmed cell death, which allows the elimination of excessively produced, improperly developed or genetically damaged cells [20], has been recognized as a possible strategy for cancer therapy. Agents that have the ability to induce apoptosis specifically in tumours have potential to be used in antitumor therapy [21]. As most of the known chemotherapeutic drugs designed to target neoplastic cells act both on cancer as well as normal cells and invariably show undesirable side effects. Hence, successful therapy should selectively eliminate the abnormal cells while leaving all normal cells functionally undisturbed. SRL that is shown in this study to induce apoptosis strongly in cancerous cell than in normal cells would fit into the category of such agents. The presence of caspase inhibitors against caspase-8 and -9 can effectively prevent SRL-induced cell apoptosis indicates that SRL-mediated cell apoptosis, likely involves both the caspase-8–regulated extrinsic pathway and the caspase-9–regulated intrinsic pathway [22], [23], [24]. The extrinsic apoptosis pathway involves ligation of the cell surface death receptors, through adaptor molecules such as Fas and FADD, resulting in recruitment of the initiator caspases-2, -8, or-10. The intrinsic apoptotic pathway is triggered by signals within the cell that target mitochondria, resulting in the release of apoptotic factors and activation of initiator caspase-9. Both the extrinsic and intrinsic pathways activate the “executioner” caspases (Caspase-3, 6 & -7) [25], [26]. The discovery that SRL-mediated cell apoptosis is associated with increased cellular levels of FasL, FADD, BID truncation, and increased PARP cleavage, all important regulators in both extrinsic and intrinsic pathways, is in keeping with the involvement of both extrinsic and intrinsic apoptotic pathways in SRL-mediated apoptosis induction.

Various plant lectins that exhibit binding specificities toward cancer-associated tumour glycans are valuable source for the development of novel anti-cancer agents [27]. Particularly interesting are the TF-binding lectins, which recognize the core 1 structure of O-linked mucin-type glycans that are commonly over expressed by up to 90% of cancers [4]. As a fungal lectin that binds specifically to the cancer-associated glycan and inhibits the growth of human breast cancer cells but has substantially less effect on normal epithelial cells, SRL has the potential to be developed as an anti-cancer agent.
